# [Retracted] KIF2C promotes the proliferation of hepatocellular carcinoma cells *in vitro* and *in vivo*

**DOI:** 10.3892/etm.2023.12095

**Published:** 2023-07-04

**Authors:** Zhenya Gao, Huanxia Jia, Fang Yu, Hongfang Guo, Baoyu Li

Oncol Lett 22:1094, 2021; DOI: 10.3892/etm.2021.10528

Following the publication of this paper, it was drawn to the Editor’s attention by a concerned reader that certain of the immunohistochemical data shown in Fig. 2A and B on p. 4 and colony formation assay data in Fig. 4A on p. 6 were strikingly similar to data that had appeared in different form in different articles published by different authors at different research institutes.

Owing to the fact that some of the data in the above article had already been published, or were under consideration for publication, prior to its submission to *Experimental and Therapeutic Medicine*, the Editor has decided that this paper should be retracted from the Journal. The authors were asked for an explanation to account for these concerns, but the Editorial Office did not receive a reply. The Editor apologizes to the readership for any inconvenience caused.

